# Supplementary material for: Active Video Games for Improving Mental Health and Physical Fitness—An Alternative for Children and Adolescents during Social Isolation: An Overview
Source: Int J Environ Res Public Health. 2021 Feb 9;18(4):1641. doi: 10.3390/ijerph18041641 (PMC7915633; doi:10.3390/ijerph18041641)
Supplement: Supplementary file 1 [file ijerph-18-01641-s001.pdf]

International Journal of
Environmental Research and Public Health

MDPI

Supplementary material

Table S1. Studies excluded

Authors, Year	Exclusion
Barkman et al., 2014	No systematic review
Barnett et al., 2011	No healthy population
Barnes & Prescott, 2018	No healthy population
Benzing, Schmidt, 2018	No systematic review
Biddiss & Irwin, 2010	No healthy population
Brilliant et al., 2019	No healthy population
Brown et al., 2016	No AVGs
Ferguson, 2015	Different outcome
Garrido et al., 2019	No AVGs
Hocking et al., 2019	No healthy population
John, Sharma, Kapanee, 2019	No AVGs
Jurdi et al., 2018	No healthy population
Li, Theng, Foo, 2016	No healthy population
Mentiplay et al., 2019	No healthy population
Norris et al., 2016	No healthy population
Nicole et al., 2014	No systematic review
Page et al., 2017	No healthy population
Perrochon et al., 2019	No healthy population
Simmich et al., 2019	No healthy population
Sun, 2015	No systematic review
Sugaya et al., 2019	No AVGs
Tomé, 2017	No AVGs
Tripette et al., 2017	No healthy population
Ufholz et al., 2019	No systematic review
Viana et al., 2020	No healthy population
Wu et al., 2019	No healthy population
Zayeni et al., 2020	No healthy population

Note: AVGs – Active video games.
